# [Retracted] MicroRNA-493 inhibits the proliferation and invasion of osteosarcoma cells through directly targeting specificity protein 1

**DOI:** 10.3892/ol.2023.14160

**Published:** 2023-11-20

**Authors:** Ming Qian, Haiyi Gong, Xinghai Yang, Jian Zhao, Wangjun Yan, Yan Lou, Dongyu Peng, Zhenxi Li, Jianru Xiao

Oncol Lett 15: 8149–8156, 2018; DOI: 10.3892/ol.2018.8268

Following the publication of this paper, it was drawn to the Editor's attention by a concerned reader that certain of the Transwell cell invasion assay data shown in Fig. 2D on p. 8152 were strikingly similar to data that had appeared in different form in a different article by different authors at different research institutes that was already under consideration for publication.

Owing to the fact that some of the data in the above article were already under consideration for publication prior to its submission to *Oncology Letters*, the Editor has decided that this paper should be retracted from the Journal. After having been in contact with the authors, they accepted the decision to retract the paper. The Editor apologizes to the readership for any inconvenience caused.

